# Study on the Influence of Rural Highway Landscape Green Vision Rate on Driving Load Based on Factor Analysis

**DOI:** 10.3390/s25020335

**Published:** 2025-01-09

**Authors:** Hao Li, Jiabao Yang, Heng Jiang

**Affiliations:** 1 School of Civil Engineering Architecture and the Environment, Hubei University of Technology, Wuhan 430068, China; 20150007@hbut.edu.cn (H.L.); 102211040@hbut.edu.cn (H.J.); 2 Key Laboratory of Intelligent Health Perception and Ecological Restoration of Rivers and Lakes, Ministry of Education, Hubei University of Technology, Wuhan 430068, China

**Keywords:** rural highway, HSV color space, green view index, visual load, factor analysis

## Abstract

The green vision rate of rural highway greening landscape is a key factor affecting the driver’s visual load. Based on this, this paper uses the eye tracking method to study the visual characteristics of drivers in different green vision environments on rural highways in Xianning County. Based on the HSV color space model, this paper obtains four sections of rural highway with a green vision rate of 10~20%, green vision rate of 20~30%, green vision rate of 30~40%, and green vision rate of 40~50%. Through the real car test, the pupil area, fixation time, saccade time, saccade angle, saccade speed, and other visual indicators of the driver’s green vision rate in each section were obtained. The visual load quantization model was combined with factor analysis to explore the influence degree of the green vision rate in each section on the driver’s visual load. The results show that the visual load of the driver in the four segments with different green vision rate is as follows: Z10~20% > Z20~30% > Z30~40% > Z40~50%. When the green vision rate is 10~20%, the driver’s fixation time becomes longer, the pupil area becomes larger, the visual load is the highest, and the driving is unstable. When the green vision rate is 40% to 50%, the driver’s fixation time and pupil area reach the minimum, the visual load is the lowest, and the driving stability is the highest. The research results can provide theoretical support for the design of rural highway landscape green vision rate and help to promote the theoretical research of traffic safety.

## 1. Introduction

According to the 2023 traffic management data approved for the study by the Hubei Provincial Highway Bureau’s science and technology project, there were 273,098 motor vehicle accidents and 62,218 deaths nationwide in 2022, with 70 percent of major traffic accidents occurring on rural roads [1].

Traffic accidents are closely related to the road traffic environment. A reasonable roadside plant landscape can actively promote the guidance of road routes, prevent glare, reduce driver fatigue, and improve driving safety. Compared with urban roads, the plant landscape environment of rural highways is disarranged and prone to extreme environments, which will in turn bring a visual load to drivers and have a negative impact on driving safety. It mainly affects the driver’s ability to recognize roads, to judge road conditions, and to perform a visual distance judgment of the road. In the traffic driving environment, people prefer a green environment, which has a certain potential for physical and mental recovery [2]. The color green is easy for the human eye to recognize and perceive as it has good brightness and contrast. The theory of stress relief proposes that a green environment (green view index) can relax the individual spirit, relieve fatigue, and make the human body full of vitality [3]. Son k et al. [4] experimentally studied white walls and green plants as visual stimuli and found that green plants stimulated participants’ alpha waves and blood pressure, and also reduced their heart rate. Jing and Zhang [5] conducted experiments in which participants looked at plants of different colors and assessed their psychological changes through psychological indicators. The results showed that green plants significantly reduced feelings of stress, restlessness, and sadness. Choi J Y et al. [6] conducted an experiment in which participants observed plants in an indoor space for 80 min according to the greening rate index (3%, 5%, 20%, and 50%), and measured the participants’ heart rate variability (HRV) and electroencephalogram (EEG) physiological response. The results showed that participants preferred a green index of 50%, and even a small amount of greenery may have a relaxing effect on people in a limited indoor space. The greenness of the road landscape is a key factor affecting drivers [7]. Li and Zhang [8] used UC-win/road to conduct a fine-grained analysis of landscape factors and suggested that appropriate plant greenness could effectively guide drivers to drive on the road and maintain their pleasant emotional state. Linghan L et al. [9] used eye tracking to explore the impact of environmental components on recovery benefits. The results showed that although there were slight differences between the physiological and psychological results, both confirmed the restorative benefits of urban green space. Partially open green Spaces with high naturalness have more positive effects than open green Spaces with high hard paved Spaces. Jiang B et al. [10] studied the influence of the highway plant landscape on driving performance, and the results showed that moderate levels of green and complexity are the best choices for driving performance. Yunwei M et al. [11] collected the driver’s eye movement and road environment data through the real vehicle test, processed the visual images using the threshold segmentation method and the target landscape area ratio method. They also expressed the driver’s sense of space closure with the spatial encirclement degree, and established the HSV color model to represent the driver’s visual perception of the mountain highway landscape environment. According to the characteristics of the normal distribution of the pupil area ratio, five levels of driver’s visual comfort were proposed, and the regression relationship between comfort and landscape information was established. It is found that there is a significant negative correlation between the road landscape information and the driver’s visual comfort. When the road landscape information is less than 0.338, the driving comfort is higher, which is conducive to driving safety. However, when the landscape information of the road area exceeds 0.644, the driving comfort decreases significantly, which is not conducive to driving safety. A reasonable landscape can make the driver’s driving safer and more comfortable. These studies all indicate that the green vision rate of plant landscape has an effect on the driving stability of drivers.

In the area of driver visual characterization, Zihui G et al. [12] used statistical methods to analyze the visual characteristics of drivers’ perception of different takeover scenarios, and explored the changes in eye movement indicators before and after the takeover request (TOR). Based on the characteristics of visual distribution and pupil change, driving behavior is analyzed to reveal the internal relationship between eye movement characteristics, takeover response, and driving control strategy. Zhifa Y et al. [13] studied the influence of factors such as a curved landscape, roadside color, landscape unit length, and enclosure degree on drivers’ psychological and physiological indexes. Haixiao W et al. [14] used the hierarchical clustering method combined with mechanical division to divide the driver’s visual field plane into six visual interest areas. Finally, the entropy weight method was used to construct a weight system of four indicators, and the concept of distraction load index was proposed. The TOPSIS method was introduced to verify the evaluation effect of distraction load index on the degree of distraction.

To summarize, previous studies on the green vision rate of plant landscape were mostly based on urban roads and garden designs, and the impact of green vision rate of rural highways on drivers’ visual characteristics has not been extensively studied. A good road driving visual environment can effectively relieve the driving pressure and fatigue of the driver, maintain a good driving state and concentration, and help the driver to accurately and quickly obtain road sign information, thus reducing the occurrence of traffic accidents. Following on from the national concept of “smooth, comfortable and beautiful”, ensuring the rationality of road visual environmental information load and the comfort and beauty of rural highway driving environment has become an important problem to be solved urgently in the field of traffic safety. Therefore, the purpose of this paper is to use the Dikablis eye tracker designed by Ergoneers GmbH in Germany to collect the driver’s eye movement index through the field driving test, analyze the relationship between the landscape greening environment and drivers’ visual load from the drivers’ visual information load and psychological characteristics, and evaluate the information load level of the road driving visual environment from the perspective of road users. It provides a theoretical basis for the comprehensive design of landscape greening rate, which is conducive to improving the visual beauty of the road environment, enhancing the cognitive efficiency of road signs, and is of great significance for building a good visual environment for rural highway driving and improving driving safety and comfort.

## 2. Materials and Methods

In this paper, the visual characteristics of drivers under different green visual environments on rural highways in Xianning County were studied by using eye tracking method. Prior to the experiment, we recruited drivers through a questionnaire between 15 July 2024 and 1 August 2024 and obtained their voluntary informed consent. The data obtained from the experiment were only used to study the driving visual characteristics of drivers on rural highways with different green vision rates in Xianning County, and to explore the impact of green vision rates on drivers’ visual load. This aimed to improve rural highway safety. The visual index data were recorded by the driver wearing eye tracker glasses in the test, and the visual data of the tested person was exported by the equipped with D-Lab(3.55.9166.0) version data analysis software. The data acquisition of the eye tracker collects the driver’s visual data at a frequency of 0.001/s, and uses excel software to process the collected visual data using the 3σ rule, removing invalid data exceeding this interval and comprehensively organize the collected data. SPSS(27.0.1.0) software was used to filter and optimize the above data, obtain the average value of the drivers’ visual data at each road section, and conduct correlation analysis on the visual data obtained in the test. Finally, factor analysis is used to explore and analyze the driving visual load.

### 2.1. Experimental Instrument

In this evaluation, the Dikablis eye tracker, designed by Ergoneers GmbH, was employed. The resolution of the pupil tracking camera is 384 pixels by 288 pixels, with an accuracy of 0.05° and a sampling rate of 60 Hz. Throughout the evaluation process, the data acquired by the eye tracker should be utilized in conjunction with its accompanying software, D-Iab, in the “.txt” format. Then, save the file format and subsequently export it to Excel for post-test processing and analysis.

### 2.2. Driving Visual Index Selection

Vision is the main source for drivers to acquire external information, and the driver’s mental activity can be understood through eye movement. Visual perception is also an important factor affecting the driver’s cognition and operation [15]. The changes in the rural highway landscape environment will affect drivers’ visual characteristics, lead to drivers’ acquisition and cognition of external information, and thus lead to traffic accidents. As an important representation of drivers’ visual perception, eye movement changes mainly have three basic manifestations: fixation, saccade, and blinking. In this study, visual parameters fixation (pupil area, fixation time) and saccade index (saccade time, saccade angle, saccade speed) were selected to represent the driver’s visual perception ability.

#### 2.2.1. Pupil Area Change

The diameter of the adult pupil is generally 2~5 mm, with an average of about 4 mm, and is circular. GAVAS R [16] and Duan [17] have concluded that changes in pupil area not only reflect human visual adaptation to changes in illumination but can also be used to characterize the magnitude of mental workload and the degree of psychological stress. The area of the pupil is automatically adjusted according to the light intensity to adjust the eye’s adaptability to light. Landscape greening rate guides the change in human emotions and controls the size of pupil area.

#### 2.2.2. Fixation Time

Fixation time refers to the duration of the driver’s fixation point remaining on the target object during the staring process, reflecting the driver’s efficiency of extracting target information [18]. Shen [19] posits that an extended duration of fixation time correlates with heightened visual load experienced during tunnel transit. When driving, the green vision rate of road landscape is inversely proportional to the fixation time, and the visual load increases with the increase in the fixation time.

#### 2.2.3. Saccade Time

Saccade time refers to the duration of the beginning and end of two consecutive fixations, which represents the time spent by the driver searching for the target [20]. Jiao F et al. [21] analyzed the driver’s saccade characteristics under different curvature conditions of urban underwater tunnel by using the driver’s saccade time and frequency, and the saccade time ratio. The results show that the average saccade time increases with the increase of radius, while the average saccade frequency decreases with the increase of radius. The driver’s scanning time is affected by the factors of the road section but is not related to the level of traffic flow.

#### 2.2.4. Saccade Angle

Saccade angle refers to the angle range of the driver’s scan in the road ahead and the surrounding environment during the driving process. Chen [22] introduced the dynamic variation in angle. This metric signifies the change in adjacent scanning angles and reflects the stability of the driver’s gaze during the driving process.

#### 2.2.5. Saccade Speed

Saccade speed reflects the speed at which the driver processes information and turns to the next target, the ratio between the saccade angle and the saccade duration, and the speed at which the driver searches the target of interest in the field of vision and the processing speed of the previous gaze information. Liang B et al. [23] collected drivers’ eye movement parameters such as fixation and saccade time, viewpoint position, pupil diameter, etc. They proposed saccade path speed indicators to evaluate drivers’ driving comfort through a questionnaire survey, finding that saccade path speed has an inverse relationship with drivers’ visual pressure.

### 2.3. Experimental Road

**Figure 1:**
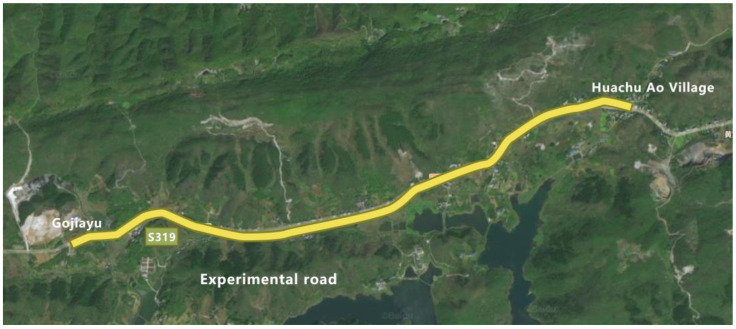
Experimental road.

According to the research characteristics and actual needs of this study, a representative secondary road in Hubei Province was selected for investigation and statistical analysis in order to gain a deeper understanding of the impact of plant landscape green vision rate on drivers’ vision. After a comprehensive consideration of geographical location, curve contour, vegetation conditions, experimental safety and other factors, we choose S319 of Chongyang County, Xianning City as the object of investigation. S319 runs through a part of Chongyang County in the east–west direction, connecting Chongyang County with surrounding areas. This section is a typical representative of a rural highway in central China, with a total length of 44 km. Through the experimental investigation on the road, this study analyzed the plant landscape environment, road alignment, traffic facilities, and geographical landforms of this rural highway, and selected a 3 km section that was suitable for this experiment. The experimental section was a two-way two-lane road with a width of 3.5 m per lane. The plant landscape of the section is rich, and the vegetation along the line is relatively dense. The linearity of the experimental road conforms to the design specifications of the corresponding grade, with fewer road intersections, pedestrians and passing vehicles, wide distribution of green rates and obvious differences in green rates, which is very suitable for exploring the impact of different green rates on driving vision. The experimental section is shown in Figure 1.

### 2.4. Driver Selection

Because the roads selected for this trial were rural highways with a high risk factor for driving, subjects with more than 3 years of driving experience were selected for this trial. According to the public security traffic control department and the Ministry of Public Security statistics, as of the end of September 2023, the country’s motor vehicle holdings amounted to 430 million vehicles, of which 330 million cars, 18.21 million new energy vehicles, 520 million motor vehicle drivers, of which 480 million car drivers, 299 million male drivers, 131 million female drivers. A total of 20 eligible drivers, including 6 females and 14 males, were selected for the test. All subjects had normal hearing, good vision levels, and no color blindness. Prior to the test, drivers were asked to ensure that they slept for the required number of hours, and to refrain from driving after consuming alcohol or stimulant drinks, as shown in Table 1.

### 2.5. Definition and Measure of Green Vision

**Figure 2:**
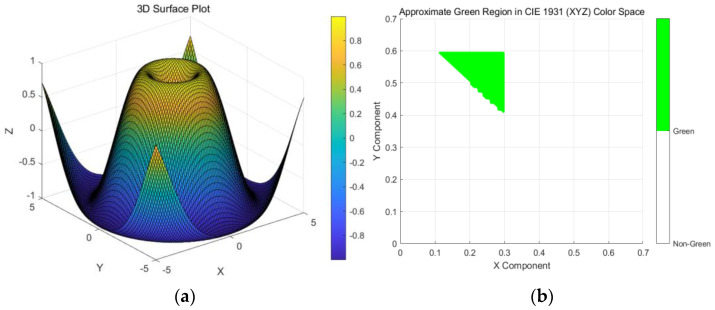
(**a**) 3D Surface Plot, (**b**) Approximate Green Region in CIE1931 (XYZ) Color Space.

In order to calculate the green vision rate of rural highway sections accurately, the HSV color space computing image model method is used in this study. The principle of calculation process is as follows:

1. Convert RGB images to HSV color space.
2. Count the number of green pixels. In the HSV color space, green is located at a point where the hue is about 60 degrees. Therefore, you can set a hue threshold range, this article set 10 to 60 degrees, to determine the green range of pixels.
3. Calculate the ratio of green pixels.
4. The boundary of green in the CIE 1931 (XYZ) color space is shown in Figure 2.

As shown in Figure 2, this is an approximation of the green region based on the CIE 1931 (XYZ) color space. The green boundary is defined as:

1. X:0.1 ≤ X ≤ 0.3.

2. Y:0.4 ≤ Y ≤ 0.6.

3. Z:0.1 ≤ Z ≤ 0.3.

**Figure 3:**
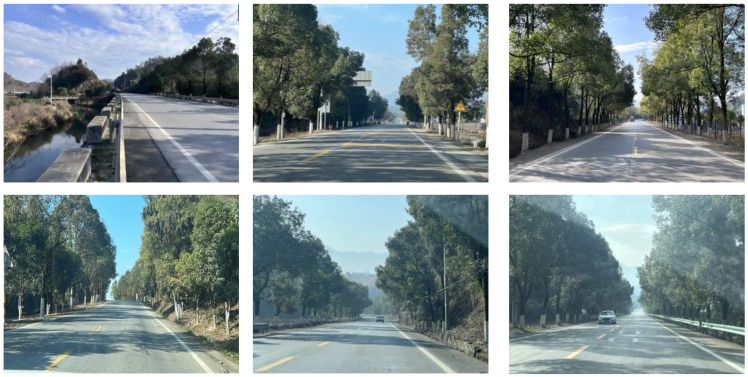
Photographs of road sections taken in the field.

Firstly, field investigation was conducted on the test road, and then photos of the road to be calculated with green vision rate were collected on the test road. I used the rear camera of the Apple 14 mobile phone to collect images through field photos, and both rear cameras were 12 million pixels. The data collection was held on 6 August 2024 during the day (9 am to 4 PM), when sunny. According to the definition of green vision rate, the acquisition height is 155 cm of the test human eye, and some photos taken are shown in Figure 3.

**Figure 4:**
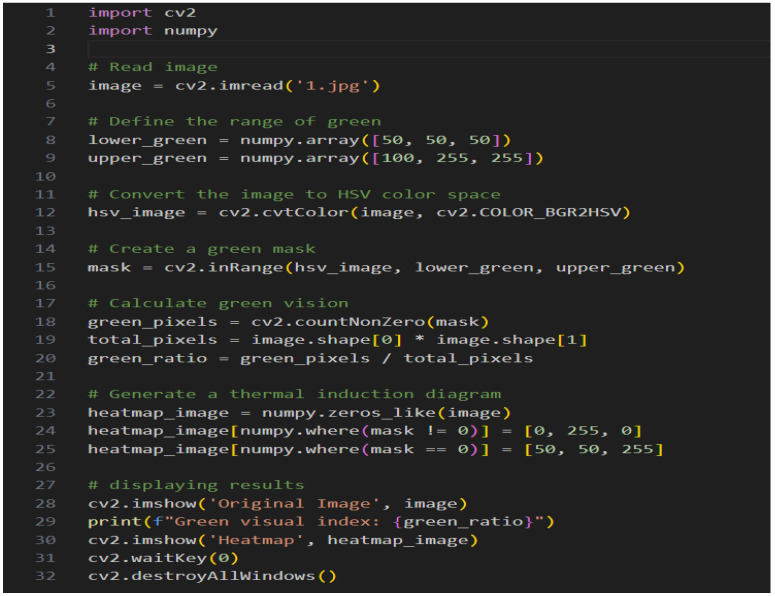
The process of measuring green ratings.

The image is automatically measured by computer and the measurement process is shown in Figure 4.

HSV color space computing image is a technique that categorizes each pixel in the entire image and labels it with its corresponding category [24], this can be achieved using neural networks, which are made to learn sample data like a human by a computer and finally analyze the results. The image measurement results are shown in Table 2.

### 2.6. Correlation Analysis Between Green Vision Rate and Driver Vision

**Figure 5:**
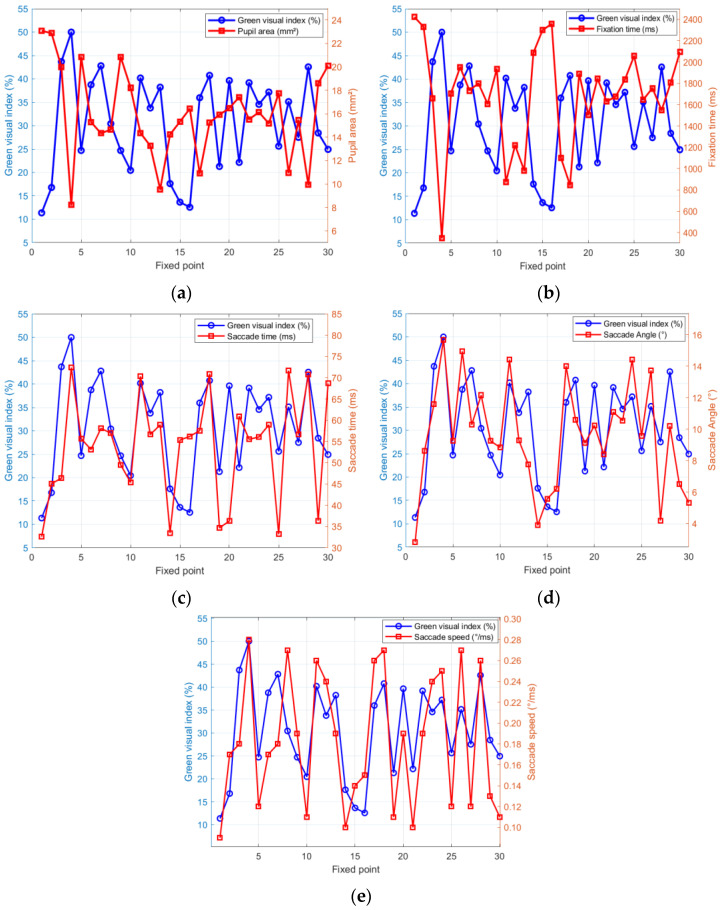
Correlation analysis diagram of green vision rate and visual index (**a**–**e**).

Based on the HSV color space model, the green vision rate of the experimental road was collected and calculated. The visual data collected by the driver wearing an eye tracker for real vehicle driving were collected. The correlation characteristics between green vision rate and driving vision were shown in Figure 5.

The relevant data results obtained through the real vehicle experiment are shown in Figure 5, in which the green vision rate has a negative linear relationship with the pupil area (a) and fixation time (b) of the driver’s vision, while the green vision rate has a significant positive linear relationship with the saccade time (c), saccade angle (d), and saccade speed (e) of the driver’s vision. The results show that the green vision rate of the rural highway plant landscape influences the driver’s vision, and the influence of different green vision rates is significant. In order to more clearly explore the impact mechanism of different green vision rates on drivers’ visual load, this study will divide the landscape green vision rates of rural highways into sections and explore the impact of different sections’ green vision rates on drivers’ visual load.

### 2.7. Green Vision Zone Division

According to the relevant research on the recognition process of plant landscapes [25], because the rural highway is complex and diverse and the green vision rate fluctuated greatly, in order to reduce the calculation error, the test section within 3000 m is selected as the visual data analysis section. The initial point at 3000 m of the road section was set as the first observation point, and the distance of 100 m was taken as the observation point interval. The test scene data of a total of 30 observation points were collected, as shown in Table 3.

According to the calculation results of green vision rate of rural highway sections as shown in Table 3, highways are divided into four sections with green vision rate of 10~20%, green vision rate of 20~30%, green vision rate of 30~40% and green vision rate of 40~50% according to the value range of green vision rate. Next, the relationship between green vision and driving vision will be explored.

## 3. Results

### 3.1. The Relationship Between Landscape Greenness and Visual Index

#### 3.1.1. Pupil Area Change

The pupil area generally reflects the driver’s efforts in focusing on a certain target, and the fatigue degree and light intensity will also affect the change in pupil area [26]. When the green vision rate of landscape is 10~20%, 20~30%, 30~40%, 40~50%, respectively, the pupil area changes after the experimenter drives the vehicle, as shown in Table 4.

**Figure 6:**
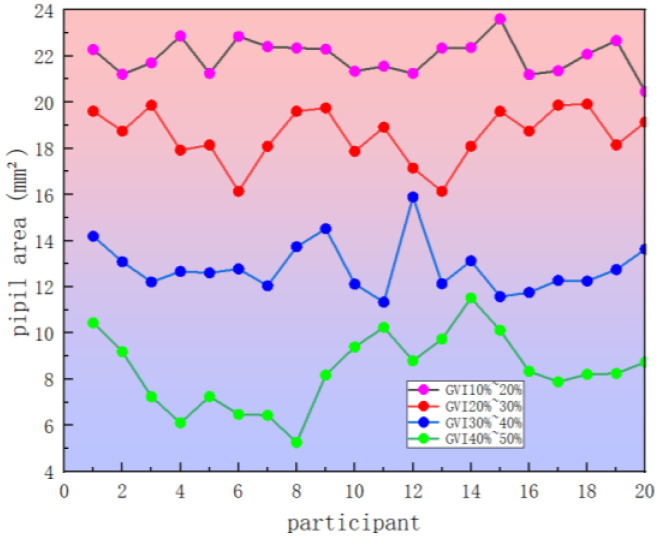
Changes in driver’s pupil area under different green vision rate.

The analysis results of the driver’s pupil area changes are shown in Figure 6. Under the four segments of landscape green vision rate, the mean pupil area of driver with the road landscape green vision rate of 40~50% is the smallest among the four segments of green vision rate. Among them, the pupil area under the landscape green vision rate of 10~20% has the largest average value. Specifically, combined with the driver’s driving experience, the single landscape environment may cause visual fatigue to the driver. The light intensity of this green vision rate section is large, and the road line of sight keeps the driver in a high state of tension. The pupils dilate as drivers choose to increase their concentration for fear of not reacting to roadside emergencies in time. The green vision rate of 40% to 50% of the scene is the opposite, the road linearity is obvious, alleviate driving fatigue, the driver in this section of the driving visual load is the lowest, the psychological and emotional relaxation of the driving stability is the highest. Liang B [26] quantifies and analyzes the visual psychological reaction of drivers in the entrance area of highway tunnels, and finds that the pupil area increases with the increase in psychological load. The results of this study are also verified.

#### 3.1.2. Fixation Time

The main information about the driver’s driving process comes from the visual search. All kinds of landscape information in the road scene and different targets will appear in the driver’s field of vision to attract attention. Drivers can fully obtain road information by looking at the landscape plants on both sides of the road, and different landscape green vision rates have obvious differences in drivers’ driving effects. The fixation time generally refers to the cumulative time of the driver’s fixation points on the plant landscape on both sides of the road, which can be used to reflect the driver’s attention distribution, and also represents the difficulty of extracting target information [18]. When the green viewing rate of the landscape was 10–20%, 20–30%, 30–40%, and 40–50%, respectively, the fixation time was changed after the experimenter operated the vehicle, as shown in Table 5.

**Figure 7:**
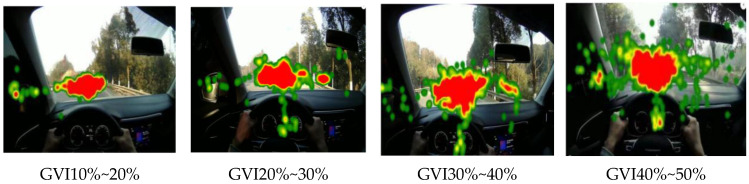
Eye movement hotspot map with different green vision rate.

**Figure 8:**
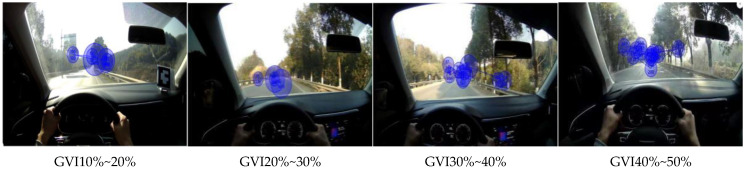
Eye trajectories with different green vision rates.

Eye movement parameters of four sections with different green vision rates were generated by eye tracker analysis software, as shown in Figure 7 and Figure 8. In the hotspot map, green to red indicates increasing attention, and red indicates the most attention. The circle in the trajectory diagram represents a strong behavior, and the larger the circle, the longer the driver’s fixation time.

**Figure 9:**
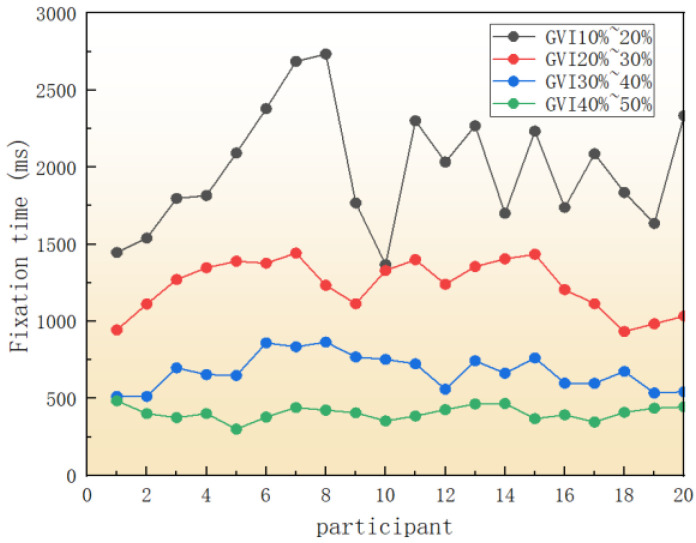
Fixation times with different green vision rates.

The analysis can be obtained by combining Table 5 and Figure 7, Figure 8 and Figure 9 above. When driving on the road with a green vision rate of 40% to 50%, the thermal map of gaze is the most widely distributed, the gaze time is short, and the attention is distributed in the center of the road and the plant landscape on both sides of the road, and the road information obtained is rich, which is conducive to the safety of road driving. When driving on the road with a green vision rate of 30% to 40%, it can be concluded that the driver’s focus is mainly on the central part of the road, in order to focus on the plant landscape on both sides, and the stability is in the middle. On the contrary, when the driver is driving at the green vision rate of 10% to 20%, his attention is distributed in the center of the road, the fixation time is greatly increased, the visual load of the driver is larger, and the driving stability is the worst. This is consistent with the research results of Shen [19], which also explains why the fixation time of the 10–20% green vision rate is higher than that of other green vision rate ranges. It is in the range of 10% to 20% green vision rate because the driver is feeling depressed and nervous, and the road environment information is reduced In order to ensure driving safety, the driver’s gaze will be more focused, the gaze time will be longer, and the attention will be highly maintained. The driver is relaxed in 40% to 50% green vision rate, and the fixation time is shorter, because the driver can clearly see the road linearity and obtain rich road information when driving in this range, and the psychological pressure is small, the driving load is low, and the driving is the most stable.

#### 3.1.3. Saccade Characteristic Analysis

Saccling can reflect the suddenness of the driver’s shift in gaze point, which is a process of searching for the target [27]. The saccade angle reflects the range of the visual field covered by the driver in a saccade process. Bingkui J [28] pointed out that the saccade time, horizontal saccade angle, and saccade speed can represent the driver’s intention. Therefore, this paper selects saccade time, horizontal saccade angle, and saccade speed to study the changes in drivers’ saccade behavior under road conditions with different green vision rates. For four road tests with different green vision rates, the saccade characteristics are shown in Table 6.

**Figure 10:**
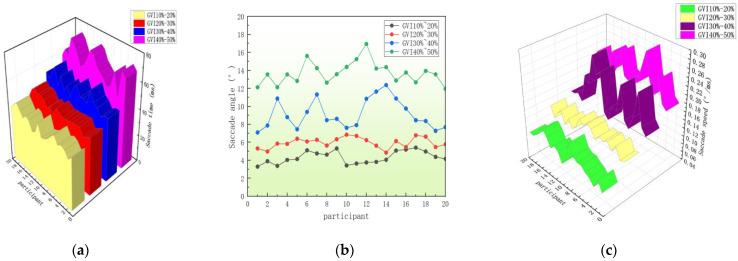
(**a**) Saccade time with different green vision rates. (**b**) Saccade angles with different green vision rates. (**c**) Saccade speed with different green vision rates.

According to the above Table 6 and Figure 10, it can be analyzed that there are obvious differences in saccades characteristics of drivers when driving on sections with different green vision rates. The driver’s saccade time, saccade angle, saccade speed, and other saccade index data all increase with the increase in green vision rate. When drivers drive in the range of 40% to 50% green vision rate, the visual load is minimal, and the psychological pressure is relieved. Combined with the driver’s feelings, drivers may be more inclined to have a wider field of vision under less pressure, and they will increase the scanning angle to observe the surrounding environment and conditions. In terms of saccade speed, drivers may make more frequent saccades because they are more likely to stay alert and look for potential hazards or changes. Drivers may increase their scanning time in order to respond quickly and accurately to their surroundings because they are more able to concentrate and less susceptible to distractions from external pressures. On the contrary, the drivers in the rate of 10% to 20%, combined with the driver’s feelings of psychological depression and tension, so that the driver focuses on the information on the road for a long time, rarely carries out scanning behavior. The scanning time, scanning angle, scanning speed are at the lowest. The driver cannot pay attention to the rear of the car information in time which easily leads to traffic accidents.

## 4. Discussion

### 4.1. Analysis of Green Vision Rate on Driving Visual Load

The service objects of rural highway landscape are drivers and passengers, and landscape greening vision has the most important impact on drivers, which is related to driving safety. Therefore, this paper compares the driving visual load value of drivers to measure the impact of green vision rate in different sections of the road landscape on driving vision. The stability of numerical value is an index to measure the volatility and dispersion of data, and it is assessed using factor analysis. Factor analysis can reduce dimension when screening and extracting potential relationships of multiple indicators. If the model results are reasonable, it can be used for comprehensive evaluation. Therefore, to represent the driving visual load, this paper intends to use visual indicators such as fixation time, pupil area, saccade time, saccade angle, and saccade speed to quantify the visual load value of the driver’s green vision rate in four different segments.

Factor analysis requires a certain correlation between the indicators, so Pearson correlation analysis is used to judge the correlation between fixation time, pupil area, saccade time, saccade angles, and saccade speed.

Visual pressure was positively correlated with fixation time and pupil area (r = 0.776, sig < 0.001). There was significant negative correlation with saccade time, saccade angle, and saccade speed (r = −0.742, −0.810, −0.754, sig < 0.001). Meet the factor analysis conditions. The test results are shown in Table 7.

The results show that when the visual pressure increases, fixation time and pupil area increase, while saccade time, saccade angle, and saccade speed decrease. Therefore, fixation time and pupil area have positive effects on visual pressure, while saccade time, saccade angle, and saccade speed have negative effects. The index of saccade time, saccade angle, and saccade speed are turned forward by using the inverse formula (NMMS), and the data are standardized using the Z-score method to carry out the factor analysis process. The common method of extracting common factors is principal component analysis. The main difference between principal component analysis and factor analysis is whether the factors are rotated. The KMO test and Bartlett sphericity test were used to determine whether the data were suitable for factor analysis, as shown in Table 8.

As can be seen from Table 8, the moderate measurement value of KMO sampling indicates that there is a high correlation among indicators, the correlation coefficient matrix is a non-unit matrix, factor analysis is effective, and common factors can be extracted from the original variables.

Table 9 shows the degree of commonality. The higher the degree of commonality, the higher the explanation degree of factor analysis to the original variable, thereby supporting the rationality of the selected index. The common degree (common variance) of these three variables is greater than or equal to 0.85, which also indicates that the extracted common factor (principal component) can well reflect the main information of the original variable, and the special factor can be ignored, as shown in Table 9.

Table 10 shows the total variance explanation. There are only five visual indicators in total. Before and after factor rotation, the variance contribution rate of the first common factor reaches 81.122% and 35.932%, respectively. This shows that it is reasonable to use the factor analysis method to extract the internal relations of the original data. As can be seen from Table 10, if the factor is not rotated, the first common factor (principal component) should be selected, the contribution rate of the square difference is 81.122%, and the corresponding eigenvalue is 4.056. The eigenvalue of the second principal component is 0.047 < 1, which does not meet the principal component selection criteria. If the factor is orthogonally rotated by the maximum variance method, the first 3 common factors are preferred, and the eigenvalues of the first 3 common factors are all greater than 1, and the cumulative variance contribution rate reaches 95.721%.

The factor load matrix before rotation is also the principal component load matrix, reflecting the degree of correlation between the common factor (principal component) and the original variable, as shown in Table 11.

As can be seen from Table 11, in the first principal component, the loads of fixation time, pupil area, saccade time, saccade angle, and saccade speed reached 0.967, 0.907, 0.887, 0.870, and 0.869, respectively, that is, all the five indicators were positive indicators when quantifying visual pressure. According to the law that fixation time, pupil area increase, saccade time, saccade angle, and saccade speed decrease when visual pressure increases, the size of visual pressure, and the contribution rates of the five indicators are successively: fixation time > pupil area > saccade time > saccade angle > saccade speed. Principal component score expression:

(1) $$F1=\frac{1}{\sqrt{4.056}}{(0.967Z}_{1}+0.907Z_{2}+0.887Z_{3}+0.870Z_{4}{+0.869Z}_{5}),$$

where $Z_{1}$, $Z_{2}$, $Z_{3}$, $Z_{4}$, $Z_{5}$ and, respectively, represent standardized original variables. The factor model is:

(2) $$Z_{1}=0.967F1$$

(3) $$Z_{2}=0.907F1$$

(4) $$Z_{3}=0.887F1$$

(5) $$Z_{4}=0.870F1$$

(6) $$Z_{5}=0.869F1$$

When factor analysis is used, a rotation transformation of factor load matrix is usually carried out to better explain the practical meaning of common factors. Table 12 shows the factor load array after rotation, and the first three common factors are in direct proportion to the five indicators. In the first common factor, the loads of fixation time and pupil area reached 0.903 and 0.736, respectively, while the loads of saccade time, saccade angle, and saccade speed were only 0.323, 0.494, and 0.302. In the second common factor, the loads of saccade time and saccade angle reached 0.858 and 0.667, while the loads of fixation time, pupil area, and saccade speed were 0.353, 0.368, and 0.374. However, in the third common factor, the saccade velocity load reaches 0.876. Combined with practical significance, the first common factor represents the visual pressure with fixation time and pupil area as the key quantitative indicators. The greater the visual pressure, the larger the fixation time and pupil area, and the smaller the saccade time, saccade angle and saccade speed. The second common factor represents the visual pressure with saccade time as the key quantitative index. The greater the visual pressure, the shorter the saccade time, the larger the fixation time and pupil area, and the smaller the saccade Angle. According to the variance interpretation table, the contribution variance of the three common factors reached 95.721%, while before rotation, the contribution rate of the first common factor (principal component) was 81.122%, which was less than that after rotation. Moreover, the factor load array after rotation showed more comprehensive content of visual pressure. Therefore, after comprehensive consideration, the first three common factors after rotation are selected as the calculation basis of the quantization model. This also shows that factor analysis is more suitable for the experimental data in this paper than principal component analysis when constructing evaluation model.

The factor model after rotation is:

(7) $$Z_{1}=0.903F1^{\prime }\;+\;0.353F2^{\prime }\;+\;0.236F3^{\prime }$$

(8) $$Z_{2}=0.736F1^{\prime }\;+\;0.368F2^{\prime }\;+\;0.561F3^{\prime }$$

(9) $$Z_{3}=0.323F1^{\prime }\;+\;0.858F2^{\prime }\;+\;0.326F3^{\prime }$$

(10) $$Z_{4}=0.494F1^{\prime }\;+\;0.667F2^{\prime }\;+\;0.405F3^{\prime }$$

(11) $$Z_{5}=0.302F1^{\prime }\;+\;0.374F2^{\prime }\;+\;0.876F3^{\prime }$$

when $F1^{\prime }$ and $F2^{\prime }$, $F3^{\prime }$ are the common factor scores after rotation.

The factor score coefficient matrix is obtained by the principal component analysis method. The negative number is caused by the transfer effect of data processing relative to the average level and does not affect the calculation result, as shown in Table 13.

The score function of the three common factors can be obtained as:

(12) $$F1^{\prime }=0.033Z_{1}+0.420Z_{2}-0.420Z_{3}-0.499Z_{4}+1.009Z_{5},$$

(13) $${F2^{\prime }=0.555Z_{1}+1.179Z_{2}+0.359Z}_{3}+0.412Z_{4}+0.271Z_{5},$$

(14) $${F3^{\prime }=0.144Z_{1}+0.389Z}_{2}+1.276Z_{3}+0.315Z_{4}-0.464Z_{5}.$$

The score of the original variable on the three common factors is calculated $F1^{\prime }$ and $F2^{\prime }$, $F3^{\prime }$. Taking the variance contribution rate corresponding to each common factor as the weight, and taking the sum of variance contribution rate as the basis for weight standardization, combined with the comprehensive linear weighting model, the calculation formula of the total driving visual load value S is finally obtained as follows:

(15) $$S=\frac{1}{0.95721}(0.3592F1^{\prime }+0.31624F2^{\prime }+0.28165F3^{\prime }).$$

The driving visual load value S calculated by factor analysis can be positive or negative, so the extreme value method is used to convert all data into the interval of [0,1], which is convenient for subsequent comparison and analysis.

(16) $$S_{i}^{*}=\left\{\begin{array}{cc} \frac{S_{i}-minS_{i}}{R_{i}}\\ R_{i}\neq 0 & i=1,2,\cdots ,x,\\ \;\;0.5\;\;\;\;\;R_{i}=0 \end{array}\right.$$

where $R_{i}$ is the max–min.

The data brought into the standardized transformation is shown in Table 14.

The driver’s visual load evaluation value uin four scenarios with different green vision rate is calculated according to the above factor synthesis model, and the load is sorted according to the principle that the smaller the value, the smaller the visual load, as shown in Table 15.

**Figure 11:**
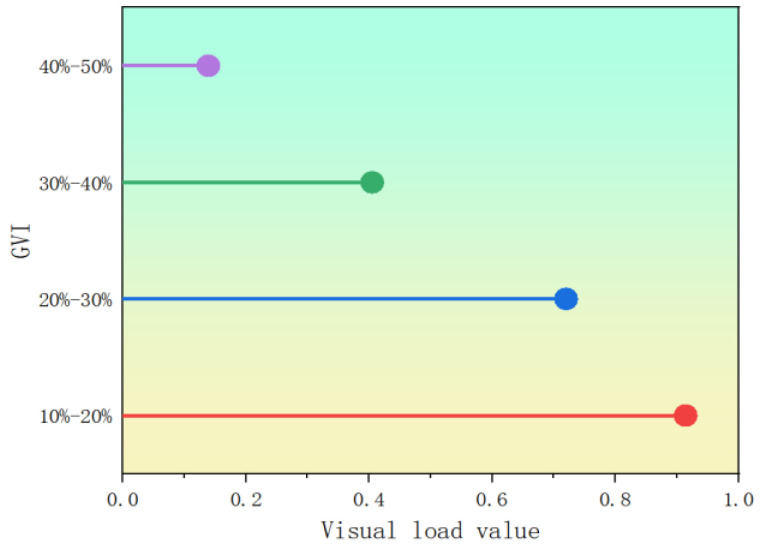
Visual load value of different green vision rate.

The comparison of visual load evaluation value under the 4-stage green vision rate is shown in Figure 11.

According to the analysis of Table 15 and Figure 11 above, the visual load of drivers is the highest in the road segment with 10% to 20% green vision rate, indicating that the road environment is relatively simple due to the sparse road landscape, and the road line of sight is fuzzy and uncertain, which is not conducive to the safety of road driving. In the green vision rate of 20~30% and 30~40% of the road, combined with the driver’s feelings, we see that the appropriate green vision rate into the driver’s field of vision can greatly alleviate the driver’s visual fatigue and visual load value. When the driver is in the green vision rate of 40~50% of the road, the driving load value reaches the lowest, and their feelings improve. This road helps to improve the cognitive ability of the driver, can make the individual spirit relax, relieve fatigue, make the human body full of vitality.

### 4.2. Comparison with Previous Studies

Based on the existing studies of plant green vision rate, it has been verified that plant green vision rate has a positive effect on people, which can induce physiological relaxation and reduce stress, tension, and anxiety. Researchers such as Son k and Choi J [4,5,6] have concluded that green can relieve visual fatigue, stress, tension, and restlessness, and the green index has the best effect at 50%. However, previous studies on plant green vision tended to focus only on urban and garden design and study the effect of green vision on the relative stillness of people, neglecting the effect of rural highway green vision on the dynamic of people.

On the other hand, according to the research of existing road landscapes, it has been established that road landscapes can guide drivers’ safety and alleviate their visual fatigue on the road. Ulrich Roger S and Jiang B [7,8,9,10] studied the influence of highway plant landscapes on driving performance. Their results showed that moderate levels of green and complexity are the best choices for driving performance. In other words, reasonable plant greenness can effectively guide drivers to drive on the road and maintain a pleasant emotional state. However, these studies do not specify a reasonable green range. Therefore, based on this study, we experimentally verified the best range of green vision rate of rural highway plant landscape by combining eye tracking and factor analysis. We determined that drivers would have the lowest visual load and the most stable driving in the range of 40–50% green vision rate.

The focus of this study is on the connectivity-oriented rural highway in Central China, rather than village-access roads or internal roads. Additionally, due to the relatively low traffic volume and number of intersections on rural highways in this region, no green vehicles were observed during the survey and experimental period. Therefore, this study did not consider the impact of traffic flow and intersections and overtaking on the experiment. Future studies could be conducted in these areas to expand existing highway safety research.

Through combining eye tracking and factor analysis, this study experimentally verified the best range of green vision rate of rural highway plant landscape, expanding the existing research on the impact of green vision rate on rural highway landscape and safety.

## 5. Conclusions

(1) In this study, a typical rural highway in central China was chosen, and the experimental sections were selected after comprehensive consideration of factors such as geographical location, curve contour, vegetation conditions, and experimental safety. The linearity of the experimental roads met the design specifications of the corresponding grade, with few road intersections, fewer pedestrians and passing vehicles, no interference from animal factors, and a wide distribution of greening rates. This location was ideal for exploring the effects of different green rates on driving vision.

(2) The quantitative model of visual load is established using factor analysis. The results show that the green vision rate of the four road sections and the visual load value of the driver are successively: Z10~20% > Z20~30% > Z30~40% > Z40~50%.

(3) The visual load of drivers under different landscape green vision rates was compared. The results show that when the green vision rate is 10~20%, the driver’s gaze time becomes longer, the pupil area becomes larger, the visual load is the highest, and the driving is unstable. When the green vision rate is 40~50%, the driver’s fixation time and pupil area are the smallest, the visual load is the lowest, and the driving stability is the highest. The results of this study can be used to analyze the visual effects of landscape green vision on drivers in different areas.

(4) The research of this paper has the following deficiencies: the study did not consider the impact of older drivers on road safety. In addition, due to the relatively small amount of traffic and intersections on rural highway in China, the study did not consider the impact of traffic flow and intersections on the experiment. In addition, the study did not consider the impact of animal presence on rural highway driving safety. Therefore, future studies could further investigate these aspects to expand existing highway safety research.

## Figures and Tables

**Table 1:** Driver’s condition.

Gender	Age			Years of Driving		
	Under 24 Years of Age	24–40 Years Old	Over 40 Years Old	3 to 5 Years	5 to 10 Years	Over 10 Years
Male	4	7	3	7	4	3
Female	2	2	2	3	2	1
Total	6	9	5	10	6	4
	20			20		

**Table 2:** Green vision measurement results.

Methods of Measurement	Original Figure	Barometer	Measurements
HSV color space computing image	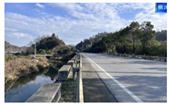	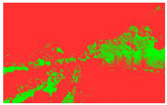	11.36%
	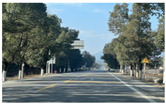	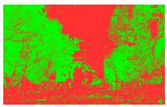	43.69%
	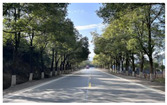	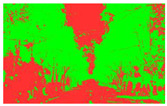	49.99%
	** 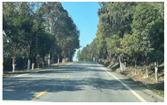 **	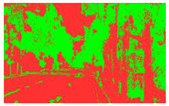	38.30%
	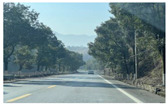	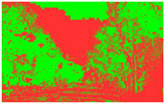	38.75%
	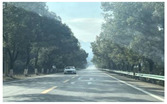	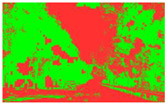	42.80%

**Table 3:** The green view rate of each scene road landscape.

Scene	GVI%	Scene	GVI%
1	11.36	16	21.86
2	16.79	17	39.19
3	43.69	18	48.65
4	49.99	19	32.07
5	24.70	20	36.19
6	38.75	21	39.47
7	42.80	22	20.66
8	30.43	23	35.88
9	24.69	24	14.07
10	20.45	25	39.35
11	40.18	26	22.11
12	33.78	27	26.84
13	38.22	28	27.90
14	17.61	29	42.28
15	13.65	30	42.30

**Table 4:** Pupil area under different green vision.

Driving Vision Index	Green View Index			
	10~20%	20~30%	30~40%	40~50%
The pupil area average (mm^2^)	21.97	18.58	12.84	8.39

**Table 5:** Fixation time under different green vision.

Driving Vision Index	Green View Index			
	10~20%	20~30%	30~40%	40~50%
Average fixation time (ms)	1989.44	1233.25	675.38	405.36

**Table 6:** Saccade characteristics under different green vision.

Driving Vision Index	Green View Index			
	10~20%	20~30%	30~40%	40~50%
Average saccade time (ms)	40.22	44.13	50.84	62.96
Average saccade angle (°)	4.33	5.96	9.13	13.70
Average saccade speed (°/ms)	0.11	0.14	0.18	0.22

**Table 7:** Visual indicator correlation.

Normalized Variable		Fixation Time	Pupil Area	Saccade Time	Saccade Angle	Saccade Speed
Correlation coefficient (r)	Fixation time	1.000	0.776	−0.742	−0.810	−0.754
	Pupil area	0.776	1.000	−0.710	−0.756	−0.684
	Saccade time	−0.742	−0.710	1.000	0.853	0.613
	Saccade angle	−0.810	−0.756	0.853	1.000	0.930
	Saccade speed	−0.754	−0.684	0.613	0.930	1.000
Significance Double tail	Fixation time		0.000	0.000	0.000	0.000
	Pupil area	0.000		0.000	0.000	0.000
	Saccade time	0.000	0.000		0.000	0.000
	Saccade angle	0.000	0.000	0.000		0.000
	Saccade speed	0.000	0.000	0.000	0.000	

**Table 8:** KMO and Bartlett sphericity test.

KMO Sample Appropriateness Measure		0.638
Bartelett sphericity test	Approximate chi-square value	582.834
	Dof	10
	Sig.	0.000

**Table 9:** Common.

Normalized Variable	Initial	Withdraw
Z-socre (Fixation time)	1.000	0.853
Z-socre (Pupil area)	1.000	0.947
Z-socre (Saccade time)	1.000	0.999
Z-socre (Saccade angle)	1.000	0.992
Z-socre (Saccade speed)	1.000	0.995

**Table 10:** Total variance interpretation.

Element	Initial Eigenvalue			Extract the Sum of Squared Loads			Rotating Load Sum of Squares		
	Total	Variance Percentage	Grand Total%	Total	Variance Percentage	Grand Total%	Total	Variance Percentage	Grand Total%
1	4.056	81.122	81.122	4.056	81.122	81.122	1.797	35.932	35.932
2	0.047	8.137	89.259	0.407	8.137	89.259	1.581	31.624	67.555
3	0.323	6.462	95.721	0.323	6.462	95.721	1.408	28.165	95.721
4	0.210	4.191	99.912						
5	0.004	0.088	100.000						

**Table 11:** Initial factor load array.

Normalized Variable	Common Factor (Principal Component 1)
Z-socre (Fixation time)	0.967
Z-socre (Pupil area)	0.907
Z-socre (Saccade time)	0.887
Z-socre (Saccade angle)	0.870
Z-socre (Saccade speed)	0.869

**Table 12:** Factor load array after rotation.

Normalized Variable	Common Factor 1	Common Factor 2	Common Factor 3
Z-socre (Fixation time)	0.903	0.353	0.236
Z-socre (Pupil area)	0.736	0.368	0.561
Z-socre (Saccade time)	0.323	0.858	0.326
Z-socre (Saccade angle)	0.494	0.667	0.405
Z-socre (Saccade speed)	0.302	0.374	0.876

**Table 13:** Factor score coefficient matrix analysis.

Normalized Variable	Common Factor 1	Common Factor 2	Common Factor 3
Z-socre (Fixation time)	0.033	0.555	0.144
Z-socre (Pupil area)	0.420	1.179	0.389
Z-socre (Saccade time)	−0.420	0.359	1.276
Z-socre (Saccade angle)	−0.499	0.412	0.315
Z-socre (Saccade speed)	1.009	0.271	−0.464

**Table 14:** Normalized processing result.

Results of Standardized Processing					
Green View Index	$Z_{1}$	$Z_{2}$	$Z_{3}$	$Z_{4}$	$Z_{5}$
10~20%	1.412	1.093	0.955	1.048	1.052
20~30%	0.243	0.541	0.554	0.613	0.509
30~40%	−0.619	−0.659	−0.133	−0.218	−0.403
40~50%	−1.036	−0.975	−1.376	−1.443	−1.158

**Table 15:** Order of visual load in different green vision zones.

	Visual Index						
Green View Index	Fixation Time (ms)	Pupil Area (mm^2^)	Saccade Time (ms)	Saccade Angle (°)	Saccade Speed (°/ms)	Visual Load Evaluation Value	Visual Load Ranking
10~20%	1989.44	22.22	40.22	4.33	0.11	0.914	4
20~30%	1233.25	20.13	44.13	5.96	0.14	0.720	3
30~40%	675.38	15.24	50.84	9.13	0.18	0.405	2
40~50%	405.36	10.04	62.96	13.70	0.22	0.139	1

## Data Availability

Data are contained within the article.
